# Absence of association between 2019‐20 influenza vaccination and COVID‐19: Results of the European I‐MOVE‐COVID‐19 primary care project, March‐August 2020

**DOI:** 10.1111/irv.12839

**Published:** 2021-01-22

**Authors:** Esther Kissling, Mariëtte Hooiveld, Mia Brytting, Ana‐Maria Vilcu, Marit de Lange, Iván Martínez‐Baz, Debbie Sigerson, Theresa Enkirch, Sylvie Belhillil, Adam Meijer, Jesus Castilla, Naoma William, AnnaSara Carnahan, Alessandra Falchi, Janneke Hendriksen, Itziar Casado, Josie Murray, Vincent Enouf, Frederika Dijkstra, Diogo F. P. Marques, Marta Valenciano

**Affiliations:** ^1^ Epiconcept Paris France; ^2^ Nivel (Netherlands Institute for Health Services Research) Utrecht The Netherlands; ^3^ Public Health Agency of Sweden Solna Sweden; ^4^ Sorbonne Université INSERM Institut Pierre Louis d'épidémiologie et de Santé Publique (IPLESP UMRS 1136) Paris France; ^5^ National Institute for Public Health and the Environment (RIVM) Bilthoven The Netherlands; ^6^ Instituto de Salud Pública de Navarra – IdiSNA – CIBERESP Pamplona Spain; ^7^ Public Health Scotland Glasgow Scotland; ^8^ National Reference Center for Respiratory Viruses Molecular Genetics of RNA Viruses Institut Pasteur UMR 3568 CNRS University of Paris Paris France; ^9^ Laboratoire de Virologie Université de Corse‐Inserm Corte France

**Keywords:** case‐control study, COVID‐19, influenza vaccination, multicentre study, SARS‐CoV‐2, test‐negative design

## Abstract

**Background:**

Claims of influenza vaccination increasing COVID‐19 risk are circulating. Within the I‐MOVE‐COVID‐19 primary care multicentre study, we measured the association between 2019‐20 influenza vaccination and COVID‐19.

**Methods:**

We conducted a multicentre test‐negative case‐control study at primary care level, in study sites in five European countries, from March to August 2020. Patients presenting with acute respiratory infection were swabbed, with demographic, 2019‐20 influenza vaccination and clinical information documented. Using logistic regression, we measured the adjusted odds ratio (aOR), adjusting for study site and age, sex, calendar time, presence of chronic conditions. The main analysis included patients swabbed ≤7 days after onset from the three countries with <15% of missing influenza vaccination. In secondary analyses, we included five countries, using multiple imputation with chained equations to account for missing data.

**Results:**

We included 257 COVID‐19 cases and 1631 controls in the main analysis (three countries). The overall aOR between influenza vaccination and COVID‐19 was 0.93 (95% CI: 0.66‐1.32). The aOR was 0.92 (95% CI: 0.58‐1.46) and 0.92 (95% CI: 0.51‐1.67) among those aged 20‐59 and ≥60 years, respectively. In secondary analyses, we included 6457 cases and 69 272 controls. The imputed aOR was 0.87 (95% CI: 0.79‐0.95) among all ages and any delay between swab and symptom onset.

**Conclusions:**

There was no evidence that COVID‐19 cases were more likely to be vaccinated against influenza than controls. Influenza vaccination should be encouraged among target groups for vaccination. I‐MOVE‐COVID‐19 will continue documenting influenza vaccination status in 2020‐21, in order to learn about effects of recent influenza vaccination.

## 1 INTRODUCTION

The end of 2019 saw the emergence of a novel severe acute respiratory syndrome coronavirus 2 (SARS‐CoV‐2), which causes coronavirus disease 2019 (COVID‐19). Europe has been heavily affected, with 14 750 809 cases and 336 422 deaths reported from Europe between 31 December 2019 and 18 November 2020.^1^ The second wave in autumn is causing increasing trends in hospital and intensive care unit admissions. ^2^ In the average winter season, hospital bed occupancy is higher, due to influenza patients.^3^, ^4^ In the 2020‐21 winter season with the ongoing COVID‐19 pandemic, it is crucial to reduce the burden on hospitals, as much as possible. Influenza vaccination, despite its sometime moderate vaccine effectiveness, is an important factor in reducing hospitalisations^5^ and is specifically important for the 2020‐21 season with SARS‐CoV‐2 circulation.

However, claims of influenza vaccination increasing COVID‐19 risk are circulating, particularly on social media. An article on influenza vaccination increasing the risk of seasonal coronaviruses^6^ has fuelled these claims, despite its results having been refuted.^7^ Additionally, ecological studies correlating influenza vaccination uptake rates and COVID‐19 mortality, published in scientific journals as articles or rapid responses to articles sustain these hypotheses.^8^, ^9^

Conversely, several articles have been published in scientific journals on the negative association or no association between influenza vaccination and COVID‐19 mortality, hospitalisation and infection,^10^, ^11^ many of which are summarised in a recent systematic literature review.^12^, ^13^, ^14^, ^15^ The authors of the latter paper recommend that further studies are carried out to validate these preliminary findings across settings.

The I‐MOVE‐COVID‐19 consortium is a network of European sites and countries, many of which are part of the I‐MOVE influenza vaccine effectiveness studies.^16^ One component of the I‐MOVE‐COVID‐19 project is a primary care multicentre study on COVID‐19.

Within this study, we aimed to measure the association between 2019‐20 influenza vaccination and COVID‐19.

## 2 METHODS

### 2.1 Study setting and study design

We conducted a multicentre test‐negative case‐control study at primary care level, in study sites in five European countries: France (FR), the Netherlands (NL), Scotland (SC), Spain (Navarra, NA) and Sweden (SE). The systems for data collection vary across sites. Briefly, ambulatory (non‐hospitalised) patients with acute respiratory infection (ARI) were swabbed for SARS‐CoV‐2 and demographic and clinical information was collected, including 2019‐20 influenza vaccination status.

Sentinel general practitioner (GP) networks in well‐established influenza surveillance systems in FR, NL and SE identified ARI patients. In SC, dedicated COVID‐19 assessment centres were used to identify patients. In NA, ARI patients were identified from data from the Navarra Health Service.

Clinical staff collected respiratory specimens face‐to‐face (NL, SE, NA, FR and SC), or specimens were received through self‐swab (SC). Information was obtained from the patient by the GP during the consultation (NL, SE and FR), by clinical staff in COVID‐19 centres (SC), by patient self‐report (SC) or through electronic medical records (Navarra).

### 2.2 Study period

The study period ranged from the ISO week of swab of the first SARS‐CoV‐2‐positive case detected by the system, until the ISO week of swab of the last SARS‐CoV‐2‐positive case up to the end of August 2020. We restricted the study until the end of August to reduce ambiguity as to which influenza vaccination to record: 2019‐20 or 2020‐21 season vaccination.

For France, the study period ended at and including ISO week 20 of swab, as from week 21‐40, influenza vaccination status was no longer collected.

### 2.3 Cases and controls

Cases were patients presenting with ARI at primary care level testing RT‐PCR positive for SARS‐CoV‐2 and controls were those testing RT‐PCR negative.

Institutionalised patients, patients with inconclusive test results, missing swab date, not agreeing to participate and those testing positive to influenza were excluded. In sites where information on influenza testing was not available, we started the study period from 1 April onwards, under the assumption that little or no influenza was circulating (one site).

### 2.4 Laboratory methods

All specimens were tested by real‐time reverse transcription quantitative (RT‐qPCR) methods.

### 2.5 2019‐20 influenza vaccination

Seasonal influenza vaccination campaigns in Europe start in early autumn.^17^ As the study period began in March 2020, after the peak of influenza in Europe, we assumed that all persons vaccinated with influenza vaccine in the study were vaccinated more than 14 days before symptom onset and could be considered fully vaccinated.

### 2.6 Primary analysis

For the primary analysis, we included only patients swabbed within 7 days of symptom onset, as PCR sensitivity is higher within this time range.^18^ Additionally, only sites with <15% of missing 2019‐20 influenza vaccination information (France, the Netherlands and Sweden) were included, to avoid a potential substantial bias due to missing data.

In the primary analysis, we carried out a complete case analysis, dropping records with missing values for covariates.

### 2.7 Secondary analysis

In a secondary analysis, we included all sites regardless of the number of days between onset of symptoms and swabbing or missing 2019‐20 influenza vaccination information (all five sites).

### 2.8 Statistical analysis

We pooled individual data and compared the odds of 2019‐20 influenza vaccination between SARS‐CoV‐2 RT‐PCR‐positive and SARS‐CoV‐2 RT‐PCR‐negative patients, with study site as a fixed effect in the model, using logistic regression. We adjusted for a priori confounders to measure adjusted odds ratios (aOR). Potential confounders included age (as measured in 5‐year age groups), sex, calendar time of swabbing, and presence of chronic conditions, including diabetes, heart disease, chronic lung disease and immunodeficiencies. The functional form (categories, continuous variable or restricted cubic spline) of 5‐year age groups and calendar time was determined using the Akaike Information Criterion (AIC).

We stratified the analysis by age group (<20 years, 20‐59 and ≥60 years) and sex.

To obtain the aOR by 5‐year age groups, we added an interaction between 2019‐20 influenza vaccination and 5‐year age groups modelled as a restricted cubic spline, adjusting for other confounders, with location of knots as defined by Harrell^19^. The number of knots was determined by the AIC, avoiding any models where the standard errors of a coefficient were greater than the coefficient themselves.

### 2.9 Sensitivity analyses

In order to test the robustness of our results, we carried out several sensitivity analyses.

In sensitivity analyses in the primary analysis, we computed the aOR between 2019‐20 influenza vaccination and COVID‐19 for other delays between onset of symptoms and swabbing: <10, <5 days, and any delay between onset of symptoms and swabbing.

In a sensitivity analysis, we used multiple imputation using chained equations to account for missing data. Briefly, we estimated missing data for 2019‐20 influenza vaccination status and covariates using multiple multivariate imputation using chained equations. We used missing at random assumptions. We used all predictors together to impute the missing values and independently analysed 20 copies of the data using 200 cycles of regression.

In the primary analysis, we did not take clustering by GP practice into account. Despite large numbers of clusters (GP practices), the number of units within clusters (patients) was low in some study sites (20% of all GP practices included <5 patients). In a sensitivity analyses, we carried out all analyses in the primary analysis using a multilevel model with GP practice as a random intercept, in order to take this clustering into account.

### 2.10 Other statistical methods

To avoid overfitting the logistic regression model, we did not attempt to measure the aOR if there were fewer than 10 cases or controls per number of parameters within the study site variable (N − 1) in the logistic regression model. If there were fewer than 10 cases or controls per number of all parameters, we carried out a sensitivity analysis using Firth's method of penalised regression.

## 3 RESULTS

Sweden, France and the Netherlands were included in the primary analysis. These countries and Scotland and Navarra were included in secondary analyses. The latter two study sites were included in secondary analyses only, as Scotland had >15% of missing 2019‐20 influenza vaccination information and data from Navarra did not include date of onset, so delay between onset of symptoms and swabbing could not be determined.

### 3.1 Primary analysis

**FIGURE 1:**
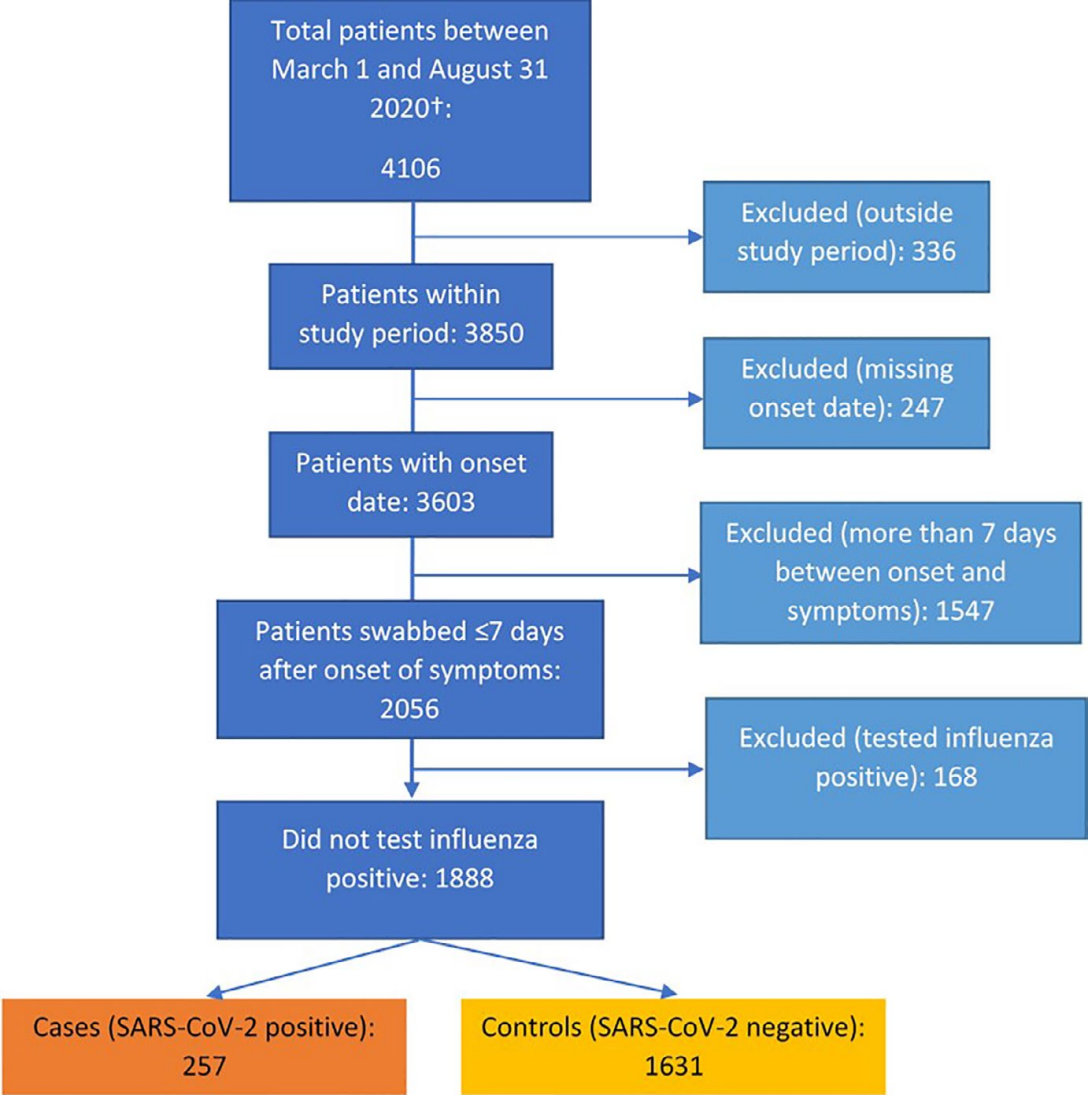
Flowchart of data exclusion of ARI patients for the pooled analysis, I‐MOVE‐COVID‐19 primary care study, primary analysis (FR, NL and SE), Europe, March‐August 2020. ARI, acute respiratory infection; FR, France; NL, The Netherlands; SE, Sweden. †Data from France were only included until 17 May 2020 (date of swab), as the data collection system after that date did not include influenza vaccination

In the primary analysis, 4106 records were received with swab dates between the 1 March 20 202 and the 31 August 2020 (see Figure 1). We included in the analysis data set 257 SARS‐CoV‐2‐positive cases and 1631 SARS‐CoV‐2‐negative controls.

**FIGURE 2:**
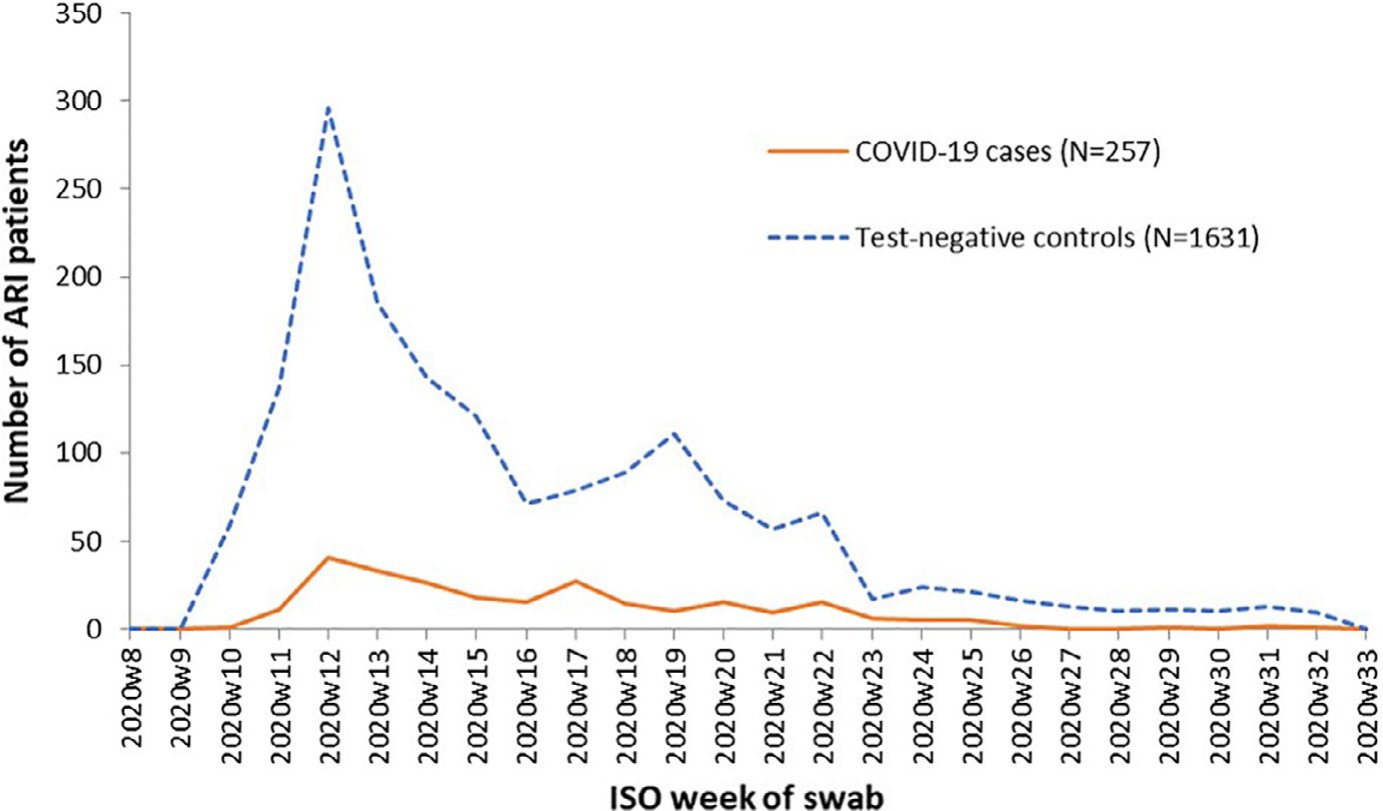
Number of ARI patients by SARS‐CoV‐2 status (test‐negative controls and SARS‐CoV‐2 cases) and week of swab, I‐MOVE‐COVID‐19 primary care study, primary analysis (FR, NL and SE), Europe, March‐August 2020. ARI, acute respiratory infection; FR, France; NL, The Netherlands; SE, Sweden

In the primary analysis, 4106 records were received with swab dates between the 1 March 2020 and the 31 August 2020 (see Figure 1). We included in the analysis data set 257 SARS‐CoV‐2‐positive cases and 1631 SARS‐CoV‐2‐negative controls (see Figure 2).

Among controls, 24% were aged <20 years, compared to 9% of cases. Fifty per cent of controls were aged 40 and over compared to 68% of cases (Table 1).

**TABLE 1:** Characteristics of SARS‐CoV‐2 cases (n = 257) and test‐negative controls (n = 1631) included in the I‐MOVE‐COVID‐19 primary care study, primary analysis (FR, NL and SE), Europe, March–August 2020

Variables	Number of SARS‐CoV‐2 cases/total n (%)	Number of test‐negative controls/total n (%)
Age groups		
0‐19	23/257 (9)	384/1630 (24)
20‐39	61/257 (24)	430/1630 (26)
40‐59	102/257 (40)	475/1630 (29)
60‐79	54/257 (21)	249/1630 (15)
80+	17/257 (7)	92/1630 (6)
Missing	0	1
Sex		
Female	149/256 (58)	994/1627 (61)
Missing	1	4
Days between onset of symptoms and swabbing		
0	11/257 (4)	80/1631 (5)
1	32/257 (12)	223/1631 (14)
2‐3	89/257 (35)	622/1631 (38)
4‐6	88/257 (34)	531/1631 (33)
7	37/257 (14)	175/1631 (11)
Seasonal influenza vaccination, 2019‐20	68/226 (30)	365/1488 (25)
Missing	31	143
≥1 chronic condition^a^	57/256 (22)	321/1623 (20)
Missing	1	8
Study site		
France	56/257 (22)	487/1631 (30)
The Netherlands	45/257 (18)	225/1631 (21)
Sweden	156/257 (61)	809/1631 (50)

Abbreviations: FR, France; NL, The Netherlands; SE, Sweden.

^a^ Out of diabetes, lung disease, immunological disease and heart disease.

The proportion of controls vaccinated with the 2019‐20 influenza vaccination was 25%, compared to 30% of cases. Overall, there was 9% of missing data for 2019‐20 influenza vaccination. 2019‐20 influenza vaccination date was known among 325 of 433 vaccinated patients (75%). The median delay between 2019‐20 influenza vaccination and onset of symptoms was 133 days among controls (interquartile range [IQR]: 109.5‐157.5) and 135 days among cases (IQR: 114‐158).

Before restricting to those swabbed within seven days of symptom onset and where delay between onset of symptoms and swabbing was known (3418/3658), the median swab delay was 5 days among cases (IQR: 3‐8) and 7 days among controls (IQR: 3‐17).

The unadjusted OR of the 2019‐20 influenza vaccination among COVID‐19 cases and controls was 1.32 (95% CI: 0.97‐1.80). The aOR was 0.93 (95% CI: 0.66‐1.32), with age as the strongest confounder (highest changes in OR when including age) (Table 2).

**TABLE 2:** Pooled odds ratio of 2019‐20 influenza vaccination among COVID‐19 cases and controls, overall and by age groups, sex and different delays between onset of symptoms and swabbing, complete case analysis. I‐MOVE‐COVID‐19 primary care study, primary analysis (FR, NL and SE), Europe, March‐August 2020

Age group	Population/analysis type^a^	N	Cases;vac/Controls; vacc	OR	CI
All ages	Crude^b^	1701	225;68/1476;361	1.32	0.97‐1.80
	Adjusted by time	1701	225;68/1476;361	1.24	0.91‐1.71
	Adjusted by time and age	1701	225;68/1476;361	0.90	0.63‐1.27
	Adjusted by time, age and chronic condition	1701	225;68/1476;361	0.91	0.64‐1.28
	Fully adjusted	1701	225;68/1476;361	0.93	0.66‐1.32
20‐59 y		950	143;32/807;170	0.92	0.58‐1.46
60+ y		367	61;35/306;178	0.92	0.51‐1.67
All ages	Males	662	93;21/569;109	0.70	0.37‐1.31
All ages	Females	1039	132;47/907;252	1.09	0.71‐1.67
Sensitivity analyses					
All ages	Symptoms ≤5 d before swab	1194	148;45/1046;252	0.92	0.60‐1.42
	Symptoms ≤7 d before swab (primary analysis)	1701	225;68/1476;361	0.93	0.66‐1.32
	Symptoms ≤10 d before swab	1975	268;78/1707;398	0.98	0.71‐1.36
	Any number of days between symptoms and onset	3182	329;87/2853;613	1.05	0.78‐1.42

Abbreviations: CI, confidence intervals; FR, France; NL, The Netherlands; OR, odds ratio; SE, Sweden.

^a^ OR adjusted by study site, time, age, sex and chronic condition unless otherwise stated.

^b^ All estimates adjusted by study site, as part of the study design.

**FIGURE 3:**
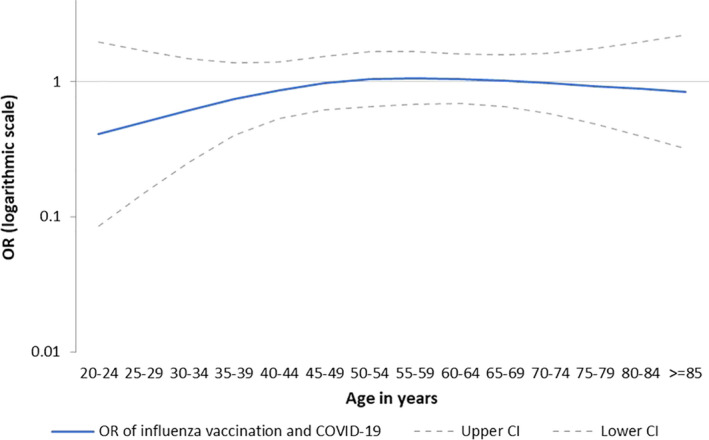
Adjusted odds ratio by 5‐y age group (*y*‐axis on logarithmic scale), I‐MOVE‐COVID‐19 primary care study, primary analysis (FR, NL and SE), Europe, March‐August 2020. CI, confidence intervals; FR, France; NL, The Netherlands; OR, odds ratio; SE, Sweden

Sample size was too small to measure the association among those aged under 20 years. The aOR was 0.92 (95% CI: 0.58‐1.46) among those aged 20‐59 and 0.92 (95% CI: 0.51‐1.67) among those aged 60 years and older. When modelling the aOR by 5‐year age group, the aOR varied between 0.41 and 1.06, but the differences were not statistically significant (Figure 3).

The aOR was 0.92 (95% CI: 0.60‐1.42) when restricting the days between onset of symptoms and swabbing to 5 or fewer days (Table 2). The aOR was 0.98 (95% CI: 0.71‐1.36) when restricting the days between onset of symptoms and swabbing to 10 or fewer days. When permitting any delay between onset of symptoms and swabbing, the aOR was 1.05 (95% CI: 0.78‐1.42).

The aOR of the imputed analysis among all ages (N = 1888) was 0.90 (95% CI: 0.64‐1.27). The imputed analysis point estimates differed by <0.05 to those of the complete case analysis for overall analyses and those stratified by age and sex (Table S1).

#### 3.1.1 Clustering by GP practice

In the primary analysis, 322 GP practices were included with a median number of three patients per GP (IQR: 1‐6) and range of 1 to 75 patients per GP practice. The aOR among all ages was 0.77 (95% CI: 0.51‐1.15). The aOR for all other estimates were lower by 0.01‐0.19 taking clustering by GP practice into account compared to the unclustered analysis (Table S2).

#### 3.1.2 Secondary analysis

The secondary analysis included France, Navarra, the Netherlands, Scotland and Sweden, presented for any delay between onset and swab, as onset date was not available in Navarra. The analyses are imputed, due to a high proportion of missing data for SC (45% after applying exclusion criteria).

We received 76 146 patients with swab dates between the 1 March 2020 and the 31 August 2020 (see Figure S1). We included in the secondary analysis data set 4739 SARS‐CoV‐2‐positive cases and 67 060 SARS‐CoV‐2‐negative controls.

The adjusted imputed OR among all ages for the association between 2019‐20 influenza vaccination and COVID‐19 was 0.86 (95% CI: 0.78‐0.95) (Table 3). This adjusted OR among all ages in the complete case analysis was 0.87 (95% CI: 0.79‐0.95) (Table S3).

**TABLE 3:** Pooled odds ratio of 2019‐20 influenza vaccination among COVID‐19 cases and controls, overall and by age groups and sex, imputed analysis. I‐MOVE‐COVID‐19 primary care study, secondary analysis (FR, NL, SE, SC and NA), Europe, March‐August 2020

Age group	Population/analysis type	N	Cases/Controls	OR	CI
All ages		71 799	4739/67 060	0.86	0.78‐0.95
20‐59 y		39 483	3138/36 345	0.88	0.77‐1.01
60+ y		16 492	897/15 595	0.86	0.73‐1.00
All ages	Males	32 928	2201/30 727	0.81	0.70‐0.95
All ages	Females	38 851	2537/36 314	0.93	0.82‐1.05

Abbreviations: CI, confidence intervals; FR, France; NA, Navarra, Spain; NL, The Netherlands; OR, odds ratio; SC, Scotland; SE, Sweden.

The adjusted imputed OR among those aged 20‐59 and 60 years and older was 0.88 (95% CI: 0.78‐0.96) and 0.86 (95% CI: 0.73‐1.00), respectively. The adjusted OR among males was 0.81 (95% CI: 0.70‐0.95) and among females was 0.93 (95% CI: 0.82‐1.05).

The imputed analysis point estimates differed by <0.02 to those of the complete case analysis for overall and stratified estimates (Table S3).

## 4 DISCUSSION

In the I‐MOVE‐COVID‐19 primary care multicentre study, there was no positive association (no increased risk) between 2019‐20 influenza vaccination and COVID‐19 cases in any analysis. This lack of positive association persisted across the primary, secondary and all sensitivity analyses, many of which had high sample size. This adds to the evidence from other studies at individual level of no positive association between 2019‐20 influenza vaccination and COVID‐19 cases.^15^

In the primary analysis including three sites, the unadjusted OR among all ages was 1.32, compared to the aOR of 0.93. This emphasises the importance of good control of confounding factors in observational studies measuring risk and preventive factors for COVID‐19. The aOR showed no significant variation across age groups. The aOR was lower among males than among females, which was consistent across analyses.

Confidence intervals in the primary analysis included the null. This was due on one hand to a reasonably low sample size (wide confidence intervals), but also due to the fact that point estimates themselves, so the anticipated biological effects, were not far from 1. For example, in the adjusted analysis among all ages, COVID‐19 cases were 10% less likely to be vaccinated with 2019‐20 influenza vaccine than controls. In secondary analyses with greater sample size, the protective effect of 2019‐20 influenza vaccination was 14% among all ages, with confidence intervals not include 1, due to a very high sample size. In both primary and secondary analyses, the point estimates suggested a minor protective effect. These results may indicate an absence of effect, or a small protective effect. Biologically, it is possible that a long‐lasting non‐specific of 2019‐20 influenza vaccination could have had a general effect on (some) virus infections.^20^ Also, an interaction with previous human coronavirus infection and 2019‐20 influenza vaccination may result in an indirect effect on SARS‐CoV‐2 infection.^21^ Replication studies and further studies on the effects of a recent influenza vaccination on COVID‐19 risk will help better understand whether there is no effect or a small protective effect of influenza vaccination on COVID‐19.

The lower aOR among males than among females, which was consistent across all analyses, may have been a spurious finding. The interaction term between sex and 2019‐20 influenza vaccination was not significant. Alternatively, we cannot exclude that unmeasured confounding differed by sex, and one of the estimates was (more) biased than the other.

As this is an observational study, we cannot exclude in general that there is unmeasured confounding in our study. The incidence of COVID‐19 varied across and within study sites, as well as 2019‐20 influenza vaccination coverage. We cannot exclude that factors other than study site, age, sex, calendar time and presence of chronic conditions were important but unmeasured confounders in the study. It may be that use of personal protective measures (eg mask use, hand washing and physical distancing), household size and frequency of leaving the house, for example, are confounders in our study. These are variables that we endeavour to collect within the I‐MOVE‐COVID‐19 study, but in practice are difficult to collect at primary care level. We plan to evaluate these factors within sub‐studies within the project. With the test‐negative design, however, we adjust for propensity to seek healthcare,^22^ which may also adjust for other unmeasured confounders. COVID‐19 studies are by definition novel and research into appropriate confounding factors at primary care level needs to be done, noting that these may be very much study dependent and time‐varying.

Taking clustering by GP into account, could be important for the aforementioned reasons of COVID‐19 incidence and 2019‐20 influenza vaccination coverage variation by GP. When taking GP into account in a multilevel model, all aOR are lower. While traditionally we expect the aOR to stay similar when accounting for clustering and the CI to be wider, changes in aOR when accounting for clustering can occur. In multilevel modelling, it is important to have a high number of clusters, and there are 322 GP practices in the primary analysis^23^. However, in this analysis, we have a low number of units by cluster (patients by GP), with 50% of GPs including three or fewer patients. The literature suggests that this low number of units by cluster may not cause an estimation effect.^24^

In the primary analysis among all ages, the aOR among those swabbed within 5 or 7 days is similar (0.92 and 0.93, respectively), but closer to the null (0.98) among those swabbed within 10 days of symptom onset. Allowing any number of days between onset of symptoms and swab increases the aOR even further to 1.05. With increasing days between onset of symptoms and swabbing, we expect to include more false‐negative COVID‐19 patients in the analysis.^18^ The change in aOR in this direction is expected, if there is a true negative association between 2019‐20 influenza vaccination and COVID‐19.

Based on this, we expect the aOR in the secondary analysis, where onset date is not available from one study site, to underestimate the association. Among all ages, the aOR in the secondary analysis allowing any number of days between onset of symptoms and swab is 0.86. Following the above logic, this may be an underestimation of the effect and the true aOR may be further away from 1.

However, we cannot be sure if the difference in aOR by varying days between onset and swabbing is due to the inclusion of false‐negative COVID‐19 cases, or entirely or in part due to other unmeasured confounding factors related to a patient getting swabbed late. Within I‐MOVE‐COVID‐19, we plan further research describing patients according to swab delay.

While a better understanding of who is swabbed early and late will help with interpretation of analyses, good data quality is key in studies to be able to make inferences. The three study sites in the primary analysis include a low proportion (<10%) of missing data for key variables. These study sites are based on well‐established sentinel influenza surveillance networks and are used to high‐quality data collection. In the secondary analysis, we include a site (Scotland) that has up to 50% of missing data for 2019‐20 influenza vaccination. Our approach of using multiple imputation to account for these missing data is an attempt to deal with missing data. While this statistical approach is useful, having fewer missing data would be preferable. In another study site in the secondary analysis (Navarra), onset date was not available. This study site increased our sample size greatly, but there is some uncertainty around the estimate as we cannot restrict to 7 days between onset of symptoms and swabbing. On the flipside, in this study sites the data quality for key variables such as vaccination status and chronic conditions was extremely high (<1% of missing data).

Systems for data collection on patients with respiratory illness have been disrupted due to the COVID‐19 pandemic. In this article, we include systems from five different countries, some with data collection limitations. Despite these limitations, the results among the primary analysis sites, with high data quality, were similar to the results among all sites, including those with issues around missing data. Additionally, some of these limitations will be improved in the near future, for example, Scotland has launched a new digital survey data collection platform^1^ for patients and clinicians to complete. In Navarra, missing data were due to an exceptional work overload due to the pandemic and have subsequently been resolved. As we move into the winter season, more I‐MOVE‐COVID‐19 study sites will be providing data. As with the sites included in this article, we will assess data quality and potential heterogeneity between sites.

Despite all the limitations of data collected during a public health emergency, we believe it is important to provide results that can contribute to the scientific evidence on risk and preventive factors for COVID‐19, in order to inform public health measures. We tried to address the limitations by conducting sensitivity analyses. All the results suggest no positive association between 2019‐20 influenza vaccination and COVID‐19 at primary care level. The results of this study underline that influenza vaccination should be encouraged among target groups for vaccination. This will help reduce the burden of health services during the COVID‐19 pandemic. In the 2020‐21 influenza season, I‐MOVE‐COVID‐19 will continue documenting influenza vaccination status, in order to repeat the analysis and learn about effects of recent influenza vaccination.

## CONFLICT OF INTEREST

No conflict of interest declared from any author/co‐author.

## AUTHOR CONTRIBUTION

**Esther Kissling:** Conceptualization (supporting); Formal analysis (lead); Funding acquisition (supporting); Methodology (equal); Writing‐original draft (lead). **Mariette Hooiveld:** Conceptualization (supporting); Funding acquisition (supporting); Investigation (equal); Methodology (supporting); Writing‐review & editing (supporting). **Mia Brytting:** Conceptualization (supporting); Funding acquisition (supporting); Investigation (equal); Methodology (supporting); Writing‐review & editing (supporting). **Ana‐Maria Vilcu:** Conceptualization (supporting); Funding acquisition (supporting); Investigation (equal); Methodology (supporting); Writing‐review & editing (supporting). **Marit de Lange:** Conceptualization (supporting); Funding acquisition (supporting); Investigation (equal); Methodology (supporting); Writing‐review & editing (supporting). **Iván Martínez‐Baz:** Conceptualization (supporting); Funding acquisition (supporting); Investigation (equal); Methodology (supporting); Writing‐review & editing (supporting). **Debbie Sigerson:** Conceptualization (supporting); Funding acquisition (supporting); Investigation (equal); Methodology (supporting); Writing‐review & editing (supporting). **Theresa Enkirch:** Conceptualization (supporting); Funding acquisition (supporting); Investigation (equal); Methodology (supporting); Writing‐review & editing (supporting). **Sylvie Behillil:** Conceptualization (supporting); Funding acquisition (supporting); Investigation (equal); Methodology (supporting); Writing‐review & editing (supporting). **Adam Meijer:** Conceptualization (supporting); Funding acquisition (supporting); Investigation (equal); Methodology (supporting); Writing‐review & editing (supporting). **Jesus Castilla:** Conceptualization (supporting); Funding acquisition (supporting); Investigation (equal); Methodology (supporting); Writing‐review & editing (supporting). **Naoma William:** Conceptualization (supporting); Funding acquisition (supporting); Investigation (equal); Methodology (supporting); Writing‐review & editing (supporting). **AnnaSara Carnahan:** Conceptualization (supporting); Funding acquisition (supporting); Investigation (equal); Methodology (supporting); Writing‐review & editing (supporting). **Alessandra Falchi:** Conceptualization (supporting); Funding acquisition (supporting); Investigation (equal); Methodology (supporting); Writing‐review & editing (supporting). **Janneke Hendriksen:** Conceptualization (supporting); Funding acquisition (supporting); Investigation (equal); Methodology (supporting); Writing‐review & editing (supporting). **Itziar Casado Buesa:** Conceptualization (supporting); Funding acquisition (supporting); Investigation (equal); Methodology (supporting); Writing‐review & editing (supporting). **Josie Murray:** Conceptualization (supporting); Funding acquisition (supporting); Investigation (equal); Methodology (supporting); Writing‐review & editing (supporting). **Vincent ENOUF:** Conceptualization (supporting); Funding acquisition (supporting); Investigation (equal); Writing‐review & editing (supporting). **Frederika Dijkstra:** Conceptualization (supporting); Funding acquisition (supporting); Investigation (equal); Methodology (supporting); Writing‐review & editing (supporting). **Diogo FP Marques:** Conceptualization (supporting); Funding acquisition (supporting); Investigation (lead); Methodology (supporting); Writing‐review & editing (supporting). **Marta Valenciano:** Conceptualization (lead); Funding acquisition (lead); Methodology (equal); Writing‐review & editing (equal).

## ETHICS APPROVAL STATEMENT

**France:** No ethical approval is needed, as stated by the French ethical review board. Document available upon request; **Sweden:** Sweden has received ethical approval on the 29 April 2020 from the ethics review authority in document “Dnr 2020‐01083”. Document available on request; **The Netherlands:** The activities carried out do not need ethical approval, as stated by the Dutch medical ethics committee, noted in the document reference “WAG/om/15/030372”. Document available on request; **Scotland:** The patient data collection, processing and analysis activities that contribute to the IMOVE project are part of usual practice in public health in compliance with national legislation, and therefore do not require ethics review nor informed consent procedures (see the following decision tool from the United Kingdom Health Research Authority http://www.hra‐decisiontools.org.uk/research/docs/DefiningResearchTable_Oct2017‐1.pdf); **Navarra:** Ethical approval was granted on the 8 May 2020 with approval number PI_2020/45. Document available upon request.

PATIENT CONSENT STATEMENT

Oral consent is required in the Netherlands, France, Sweden and Scotland. Patient informed consent procedures are available upon request. Informed consent is not required in Navarra.

PERMISSION TO REPRODUCE MATERIAL FROM OTHER SOURCES

No reproduced material is included.

### PEER REVIEW

The peer review history for this article is available at https://publons.com/publon/10.1111/irv.12839.

## Supporting information

Supplementary Material

## Data Availability

Data available on request due to privacy/ethical restrictions: The data that support the findings of this study are available on request from the corresponding author. The data are not publicly available due to privacy or ethical restrictions.

## Footnotes

^1^
https://covid19surveys.publichealthscotland.scot/
