# Supplementary material for: Absence of association between 2019‐20 influenza vaccination and COVID‐19: Results of the European I‐MOVE‐COVID‐19 primary care project, March‐August 2020
Source: Influenza Other Respir Viruses. 2021 Jan 22;15(4):429–38. doi: 10.1111/irv.12839 (PMC8013620; doi:10.1111/irv.12839)
Supplement: Supplementary file 1 — Supplementary Material [file IRV-15-429-s001.docx]

Supplementary information

**Figure S1.** Flowchart of data exclusion of ARI patients for the pooled analysis, I-MOVE-COVID-19 primary care study, secondary analysis (FR, NA, NL, SE, SC), March–August 2020

Total patients between March 1 and August 31 2020†:
76137

Cases (SARS-CoV-2 positive): 4739

Excluded (outside study period): 426

Is institutionalised: 71982

Controls (SARS-CoV-2 negative): 67051

Patients within study period: 75711

Excluded (is institutionalised): 3729

Excluded (tested influenza positive): 192

Did not test influenza positive: 71790

**Any delay between onset of symptoms and swabbing
(FR, NA, NL, SE, SC)**

FR: France; NL: The Netherlands; SE: Sweden; SC: Scotland; NA: Navarra, Spain.

† Data from France was only included until May 17 2020 (date of swab), as the data collection system after that date did not include influenza vaccination. Data from Navarra were only included from 1^st^ of April 2020 (date of swab), to avoid including potential influenza positive cases.

**Table S1.** Pooled imputed odds ratio of 2019–20 influenza vaccination among COVID-19 cases and controls, overall and by age groups, sex and different delays between onset of symptoms and swabbing. I-MOVE-COVID-19 primary care study, primary analysis (FR, NL, SE), Europe, March–August 2020

| **Age group** | **Population / analysis type** | **N** | **Cases / Controls** | **OR** | **CI** |
| --- | --- | --- | --- | --- | --- |
| All ages |  | 1881 | 257/1624 | 0.91 | 0.65-1.28 |
| 20-59 years |  | 1063 | 163/900 | 0.93 | 0.58-1.47 |
| 60+ years |  | 412 | 71/341 | 0.91 | 0.49-1.70 |
| All ages | Males | 737 | 107/630 | 0.71 | 0.37-1.36 |
| All ages | Females | 1139 | 149/990 | 1.08 | 0.71-1.66 |
| All ages | Symptoms ≤4 days before swab | 1302 | 162/1140 | 0.92 | 0.60-1.42 |
|  | Symptoms ≤7 days before swab | 1881 | 257/1624 | 0.91 | 0.65-1.28 |
|  | Symptoms ≤10 days before swab (primary analysis) | 2187 | 307/1880 | 0.95 | 0.68-1.32 |
|  | Any number of days between symptoms and onset | 3649 | 384/3265 | 1.03 | 0.77-1.37 |

FR: France; NL: The Netherlands; SE: Sweden; OR: odds ratio; CI: confidence intervals.

**Table S2.** Pooled odds ratio of 2019–20 influenza vaccination among COVID-19 cases and controls, overall and by age groups, sex, and by different delays between onset of symptoms and swabbing, adjusted for clustering by GP practice. I-MOVE-COVID-19 primary care study, primary analysis (FR, NL, SE), Europe, March–August 2020

| **Age group** | **Population / analysis type** | **N** | **Cases;vacc / Controls; vacc** | **OR** | **CI** |
| --- | --- | --- | --- | --- | --- |
| All ages | Fully adjusted | 1694 | 225;68 / 1469;360 | 0.77 | 0.51-1.15 |
| 20-59 years | Fully adjusted | 945 | 143;32 / 802;169 | 0.80 | 0.47-1.36 |
| 60+ years | Fully adjusted | 367 | 61;35 / 306;178 | 0.84 | 0.41-1.72 |
| All ages | Males (fully adjusted) | 659 | 93;21 / 566;109 | 0.69 | 0.33-1.40 |
| All ages | Females (fully adjusted) | 1035 | 132;47 / 903;251 | 0.91 | 0.56-1.48 |
| All ages | Symptoms ≤4 days before swab | 1190 | 148;45 / 1042;252 | 0.79 | 0.48-1.31 |
|  | Symptoms ≤7 days before swab | 1694 | 225;68 / 1469;360 | 0.77 | 0.51-1.15 |
|  | Symptoms ≤10 days before swab (primary analysis) | 1968 | 268;78 / 1700;397 | 0.82 | 0.57-1.18 |
|  | Any number of days between symptoms and onset | 3173 | 329;87 / 2844;611 | 0.92 | 0.67-1.28 |

FR: France; NL: The Netherlands; SE: Sweden; OR: odds ratio; CI: confidence intervals.

**Table S3.** Pooled odds ratio of 2019–20 influenza vaccination among COVID-19 cases and controls, overall and by age groups and sex, complete case analysis. I-MOVE-COVID-19 primary care study, primary analysis (FR, NA, NL, SC, SE), Europe, March–August 2020

| **Age group** | **Population / analysis type** | **N** | **Cases;vacc / Controls; vacc** | **OR** | **CI** |
| --- | --- | --- | --- | --- | --- |
| All ages |  | 65437 | 4630;766 / 60807;13268 | 0.87 | 0.79-0.95 |
| 20-59 years |  | 36406 | 3067;265 / 33339;3734 | 0.88 | 0.77-1.01 |
| 60+ years |  | 15225 | 869;462 / 14356;8469 | 0.86 | 0.74-1.01 |
| All ages | Males | 30177 | 2153;311 / 28024;5759 | 0.82 | 0.70-0.96 |
| All ages | Females | 35260 | 2477;455 / 32783;7509 | 0.91 | 0.80-1.03 |

FR: France; NL: The Netherlands; SE: Sweden; SC: Scotland; NA: Navarra, Spain; OR: odds ratio; CI: confidence intervals.
